# The Role of NDRG1 Expression in Vasculogenic Mimicry of High-grade Gliomas

**DOI:** 10.7150/jca.100458

**Published:** 2024-10-28

**Authors:** Haoxiaoyu Yang, Xuqiu Qin, Ruihao Zhang, Jiayi Miao, Jiangli Cui, Yi Li, Xingyu Miao

**Affiliations:** 1 Department of Neurosurgery, Shaanxi Provincial People's Hospital, Xi'an, China.; 2 Department of Lung Cancer Surgery, Tianjin Medical University General Hospital, Tianjin, China.; 3 Xi'an Medical University, Xi'an, China.

**Keywords:** NDRG1, Vasculogenic mimicry, Glioma, MVD, GBM

## Abstract

**Background:** Glioma is one of the most common primary malignant tumors of the central nervous system, and the prognosis is getting worse with the improvement of tumor grade. In recent years, with the in-depth study of the disease, researchers have found that microvascular mimicry seems to be the key factor of poor prognosis, but its specific mechanism has not been thoroughly studied, and the expression of microvascular mimicry in high-grade gliomas is closely related to the expression of NDRG1 gene. This research project is expected to control the occurrence and development of HGG by exploring the factors that affect the expression of NDRG1 gene.

**Methods:** Retrospective RNA-seq data analysis was performed on Chinese Glioma Genome Atlas (CGGA) database and glioma patients from TCGA. Subsequently, 7 cases of CNSWHO3 grade patients and 36 cases of CNSWHO4 grade patients were selected based on postoperative pathological results, and clinical information of the patients was collected. Immunohistochemical staining of relevant proteins was conducted on patient tissues, and the results were recorded after double immunostaining scoring for each slide. Statistical analysis of the data was performed using relevant statistical methods.

**Results:** The analysis of two databases shows that NDRG1 is enriched in HGG samples. NDRG1 is an independent risk factor for overall survival (OS) in glioma patients, and plays an important role in promoting VM in glioma patients. Different protein immunohistochemistry double immunostaining scores show that the expression levels of NDRG1 and Vimentin are correlated with tumor grade in HGG patients. The expression of VM in samples is closely related to the expression level of NDRG1 (P=3.98E-04) and tumor CNSWHO grade (P=0.019). MVD in HGG patients is related to NDRG1 expression (P=0.035). The rest of the immunohistochemistry scores are not statistically significant (P>0.05). There is a trend of correlation between Vimentin expression and MVD in CNSWHO grade 4 HGG patients (P=0.07).

**Conclusions:** NDRG1 exerts adverse effects on the CNSWHO grading of HGG by regulating the expression of VM and MVD in HGG patients, as well as the tumor EMT process.

## Introduction

Glioblastoma, as one of the most common primary malignant tumors of the central nervous system, has long been widely recognized in the medical field due to its highly invasive nature and treatment resistance. Despite significant advancements in the diagnosis and treatment of glioblastoma in recent years, driven by the rapid development of multidisciplinary fields such as medical imaging, neurosurgery, pathology, and molecular biology, its high mortality and disability rates continue to make it a challenging research topic in the field of neurological diseases^1^. In the field of glioma research, angiogenesis and its regulatory mechanisms have always been a hot topic. The traditional view holds that tumor angiogenesis mainly relies on the formation of new blood vessels by endothelial cell proliferation^2^-^4^. However, in recent years, more and more evidence suggests that in addition to endothelial cell-derived vessels, there is also a non-endothelial cell-derived mechanism of blood vessel formation, known as vasculogenic mimicry (VM). At the end of the 20th century, Maniotis *et al.* first proposed the concept of VM in the study of melanoma, which was a significant challenge to the traditional theory of angiogenesis^5^. Prior to this, scientists generally believed that the growth and spread of tumors mainly relied on neovascularization formed by endothelial cells to provide the necessary nutrients and oxygen for the tumor. VM in gliomas is a three-dimensional structure similar to blood vessels formed by tumor cells mimicking endothelial cells and surrounding basement membrane through cell-to-cell connections. It can provide nutrition and oxygen support for tumors without the involvement of endothelial cells, promoting tumor growth and spread^6^. Currently, the diagnostic criteria for VM mainly include: A absence of endothelial cells in the VM lumen; B detection of tumor cells within the lumen; C positive PAS staining and negative CD31 staining in the lumen; D detection of red blood cells within the lumen^7^, ^8^.

Currently, many hypotheses have been proposed in academia regarding the mechanism of VM formation in tumors, and numerous key gene loci have been identified. Among them, NDRG1 has become a hot gene in the process of tumor VM formation^9^, and currently, there is little research on how the NDRG1 gene promotes the VM process in HGG.

The NDRG1 gene is located on human chromosome 8q24.2, containing multiple exons and introns. Its encoded protein is a cytoplasmic protein with a molecular weight of 43kDa, belonging to the α/β hydrolase superfamily. This protein has various biological functions, including cell cycle regulation, apoptosis, cell migration, and invasion^10^, ^11^. Recent articles have reported abnormal expression of NDRG1 in various cancers, and the expression levels of this gene in different tumors are not completely consistent, even showing opposite tumorigenic effects in different tumors. The NDRG1 gene has a significant tumor suppressive effect in tumors such as gastric cancer, colorectal cancer, ovarian cancer, prostate cancer, and renal cancer. However, in other tumors such as hepatocellular carcinoma, oral squamous cell carcinoma, nasopharyngeal carcinoma, osteosarcoma, and non-small cell lung cancer, upregulation of NDRG1 is often associated with poor tumor outcomes^12^. Literature also suggests that abnormal expression of NDRG1 is related to poor prognosis in HGG (especially GBM) patients^13^. Related studies have shown that NDRG1 gene affects the prognosis of GBM mainly by affecting the NF- κ B signal pathway and then affecting the expression of VM in tumor. Therefore, this research team involved the following immunohistochemical experiments to explore the relationship between NDRG1 gene expression and VM in GBM, in order to explore a new strategy for the treatment of GBM.

## Methods

### Ethical statement and specimen source

All patients involved in the study signed informed consent and allowed researchers to use their tumor samples for research. All studies involved in this research have been approved and authorized by the Review Committee and Ethics Committee of Shaanxi Provincial People's Hospital. All specimens of glioma patients were obtained from 43 patients who underwent craniotomy surgery for tumor resection at Shaanxi Provincial People's Hospital from May 2019 to October 2023, and postoperative pathological results met the diagnostic criteria of HGG. Clinical information of the patients was collected. All tumor samples were fixed with formalin, embedded in paraffin, and subsequently sectioned and read by two experienced pathologists. The high-grade glioma specimens involved in this study were histologically classified according to the WHO 2021 classification criteria for central nervous system tumors. Among them, there were 7 cases of CNS WHO grade 3 and 36 cases of CNS WHO grade 4, all of which were GBM.

Table 1 summarizes the general clinical data of 43 patients in this study, including 31 males (72.1%) and 12 females (27.9%). The average age at the time of surgery was 60 years (range 25-82 years). Seven cases (16.3%) were classified as CNS WHO3 grade, while 36 cases (83.7%) were classified as CNS WHO4 grade.

### Immunohistochemistry experiment

All tumor specimens were obtained intraoperatively and promptly fixed in 10% neutral formalin solution at room temperature before being embedded in paraffin. Prior to the commencement of the study, paraffin blocks of the specimens were sectioned into slices with a thickness of approximately 5 micrometers. These slices were utilized to investigate the expression of proteins relevant to the study samples. The specific experimental procedure entailed sequential immersion of the slices in environmentally friendly xylene (10 minutes each for stages I, II, and III) and absolute ethanol (5 minutes each for stages I, II, and III), followed by a final rinse in distilled water. The slices were immersed in EDTA buffer (pH9.0), heated in microwave oven for 8 min-cease-fire for 8 min-7min at medium and low heat, then cooled naturally and washed with PBS (PH7.4) for 3 times (each time 5min). The slices were put into 3% hydrogen peroxide solution and incubated at room temperature for 25 minutes and washed for 3 times (5min each time).3% BSA was evenly added to cover the tissue in the immunohistochemistry circle, and incubated at room temperature for 30 minutes. Gently remove the blocking solution, and add primary antibodies against VEGFA (rabbit monoclonal antibody, GB15165, dilution 1:250; Wuhan Servicebio Technology Co., Wuhan, China), vimentin (rabbit monoclonal antibody, GB111308, dilution 1:1500; Wuhan Servicebio Technology Co., Wuhan, China), N-cadherin (mouse monoclonal antibody, GB12135, dilution 1:1500; Wuhan Servicebio Technology Co., Wuhan, China), E-cadherin (mouse monoclonal antibody, GB12083, dilution 1:5000; Wuhan Servicebio Technology Co., Wuhan, China), NDRG1 (rabbit monoclonal antibody, #5482, dilution 1:500; CST, USA) on the sections. The sections were laid flat in a humid box and incubated overnight at 4°C. The slides were placed in PBS (PH 7.4) and shaken on a decolorization shaker for 3 times, each time for 5 minutes. After slightly drying the sections, add HRP-conjugated goat anti-rabbit IgG secondary antibody (GB23303, dilution 1:200; Wuhan Servicebio Technology Co., Wuhan, China) and HRP-conjugated goat anti-mouse IgG secondary antibody (GB23301, dilution 1:200; Wuhan Servicebio Technology Co., Wuhan, China) corresponding to the primary antibodies in the immunohistochemistry circle, cover the tissue, and incubate at room temperature for 50 minutes. Then the slides were placed in PBS (PH 7.4) and shaken on a decolorization shaker for 3 times, each time for 5 minutes. DAB chromogenic solution (controlled under microscope), rinse with tap water to stop coloring. Hematoxylin re-staining, tap water rinsing, differentiation liquid differentiation, blue solution turning blue, running water rinsing. The slices were successively dehydrated by alcohol gradient dehydration, n-butanol and xylene transparent, dried and sealed. The results were observed and interpreted under white light microscope.

### Double staining of CD31 and PAS

Due to the positive CD31 staining in endothelium-dependent blood vessels and negative CD31 staining in vascular mimicry, as well as positive PAS staining, the research team conducted detection using a dual staining method of CD31 and PAS to investigate the two microcirculation patterns of tumors. Briefly, tumor specimens with a thickness of approximately 5μm were cut from paraffin-embedded tumor blocks and subjected to standard immunohistochemical staining. The process involved the use of antibodies against CD31 (rabbit monoclonal antibody, GB113151, dilution 1:500; Wuhan Service biotechnology CO., Wuhan, China) and HRP-labeled goat anti-rabbit IgG secondary antibody (GB23303, dilution 1:200; Wuhan Service biotechnology CO., Wuhan, China). The sections were then rinsed with distilled water for 5 minutes, incubated with periodic acid-Schiff (PAS) for 15 minutes, and counterstained with Mayer's hematoxylin for 1 minute. Finally, CD31 and PAS signals in each section were examined under a light microscope and recorded for analysis.

### Immunohistochemical evaluation

Immunohistochemical staining results were evaluated at a magnification of 600 times under light microscopy. Three random areas were selected on each slide, and a dual immunostaining scoring method was used to score NDRG1, E-cad, N-cad, vimentin, and VEGFA proteins. The average score was then taken as the total staining score of the target protein in that slide. The specific scoring method is as follows: first, scoring is based on the staining intensity of the target protein in the microscopic field of view: 0 (negative), 1 (weak), 2 (moderate), 3 (strong). Secondly, scoring is based on the proportion of positive cells in the microscopic field of view for the target protein: 0 (0%-5%), 1 (5%-25%), 2 (25%-50%), 3 (50%-75%), 4 (75%-100%). Finally, the scores from these two parts are added together to obtain the total staining score. A total staining score of 0-5 points is classified as the low expression group, while a score of 6-7 points is classified as the high expression group.

CD31 and PAS double staining was used to distinguish tumor microvessels and VM. Microvessel density (MVD) is evaluated by counting CD31-positive channels in 5 randomly selected fields at a magnification of 300 times, and the presence of VM expression is observed, followed by calculating the average count.

The interpretation of all staining results and data entry were independently evaluated by two experienced pathologists using a double-blind method.

### Data analysis

In order to explore the correlation between the expression levels of NDRG1 gene, EMT-related proteins, VEGFA expression levels, VM expression, and tumor CNS WHO grading, we used a continuous corrected chi-square test. Additionally, to further investigate the correlation between the immunohistochemical scoring of EMT-related proteins such as NDRG1, N-cad, E-cad, VEGFA, Vimentin in specimens, tumor CNS WHO grading, and clinical characteristics of patients, we employed Pearson correlation coefficient analysis. In all statistical tests, we set P<0.05 as the significance level. In the research process of this study, in order to accurately and visually display the data analysis results, we used a variety of professional software including SPSS 25.0, Origin 2018, RStudio 2023.12.1+402, Adobe Illustrator 2024 for data processing and chart drawing.

## Results

### NDRG1 is upregulated in gliomas

**Figure 1:**
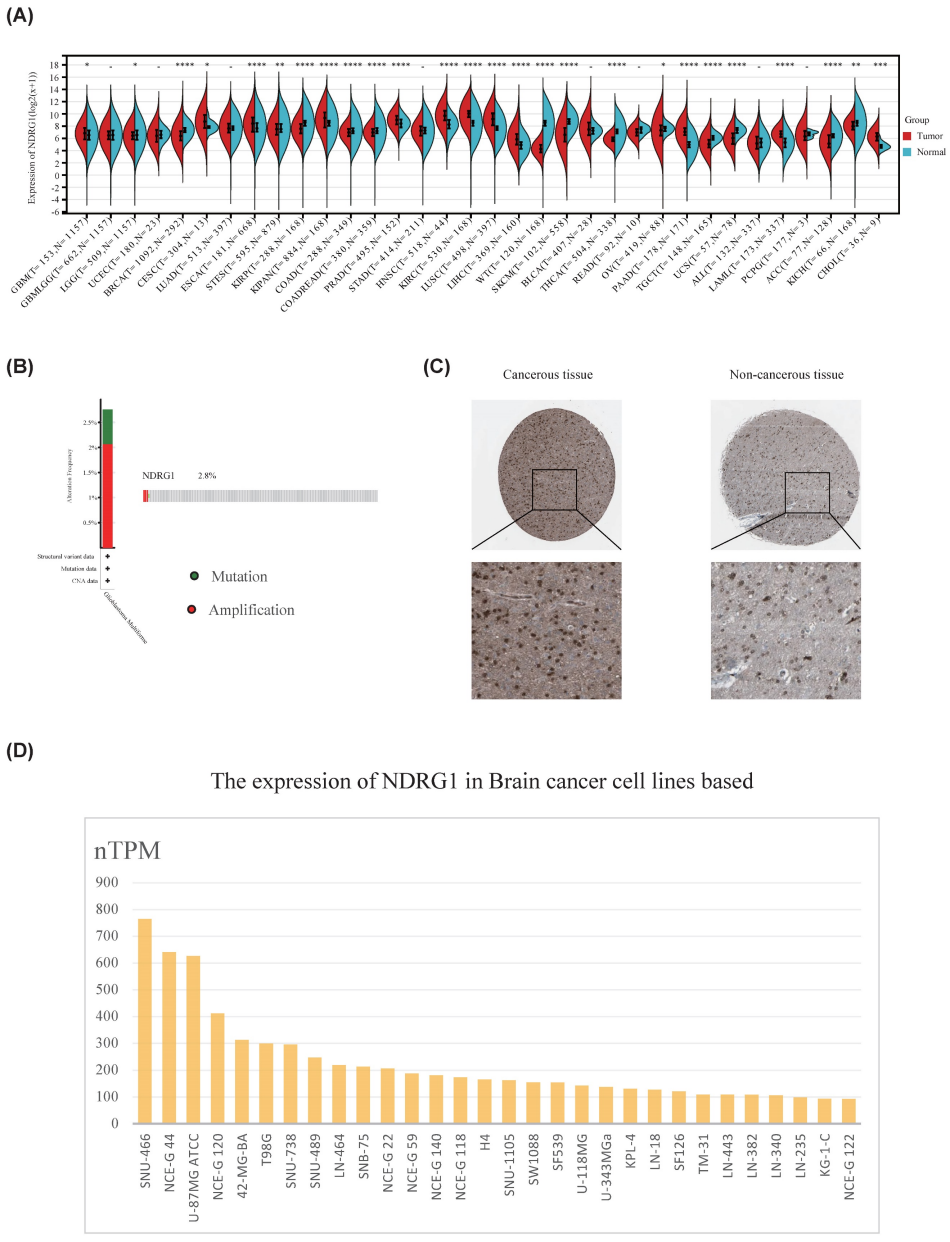
Expression of NDRG1 in glioma. A Standardized pan-cancer dataset in the UCSC database (https://xenabrowser.net/): TCGA TARGET GTEx (PANCAN, N=19131, G=60499), showing the expression of NDRG1 in 34 types of tumors and normal tissues; B Glioma database based on TCGA, analyzing gene mutation status of NDRG1 through cBioPortal; C Immunohistochemical detection of NDRG1 protein expression in glioma tissues and adjacent brain tissues in the Human Protein Atlas database; D Expression of NDRG1 in 30 brain cancer cell lines in the Human Protein Atlas database.

In order to explore the role of NDRG1 in glioma, we first studied the expression of NDRG1 in 34 types of tumors and normal tissues through the standardized pan-cancer dataset in the UCSC database (https://xenabrowser.net/). The results showed that NDRG1 was upregulated in glioma, cervical squamous cell carcinoma, mixed renal cell carcinoma, pancreatic cancer, and other tumors (Figure 1A). Furthermore, we analyzed the mutation status of NDRG1 in glioma using the cBioPortal database, and the results showed that NDRG1 gene had genetic alterations in 2.8% of glioma patients, mainly manifesting as mutations and amplifications (Figure 1B).In order to further clarify the expression of NDRG1 in gliomas, we used the analysis results in the HPA database to show that the protein expression of NDRG1 in glioma tissues was significantly increased compared to paired normal tissues (Figure 1C). To further validate the research results, we utilized the HPA database to analyze the expression of the NDRG1 gene in various brain cancer cell lines, and the results showed that NDRG1 was upregulated in multiple brain cancer cell lines (Figure 1D).

### NDRG1 is abundantly expressed in gliomas with malignant prognostic molecular markers

**Figure 2:**
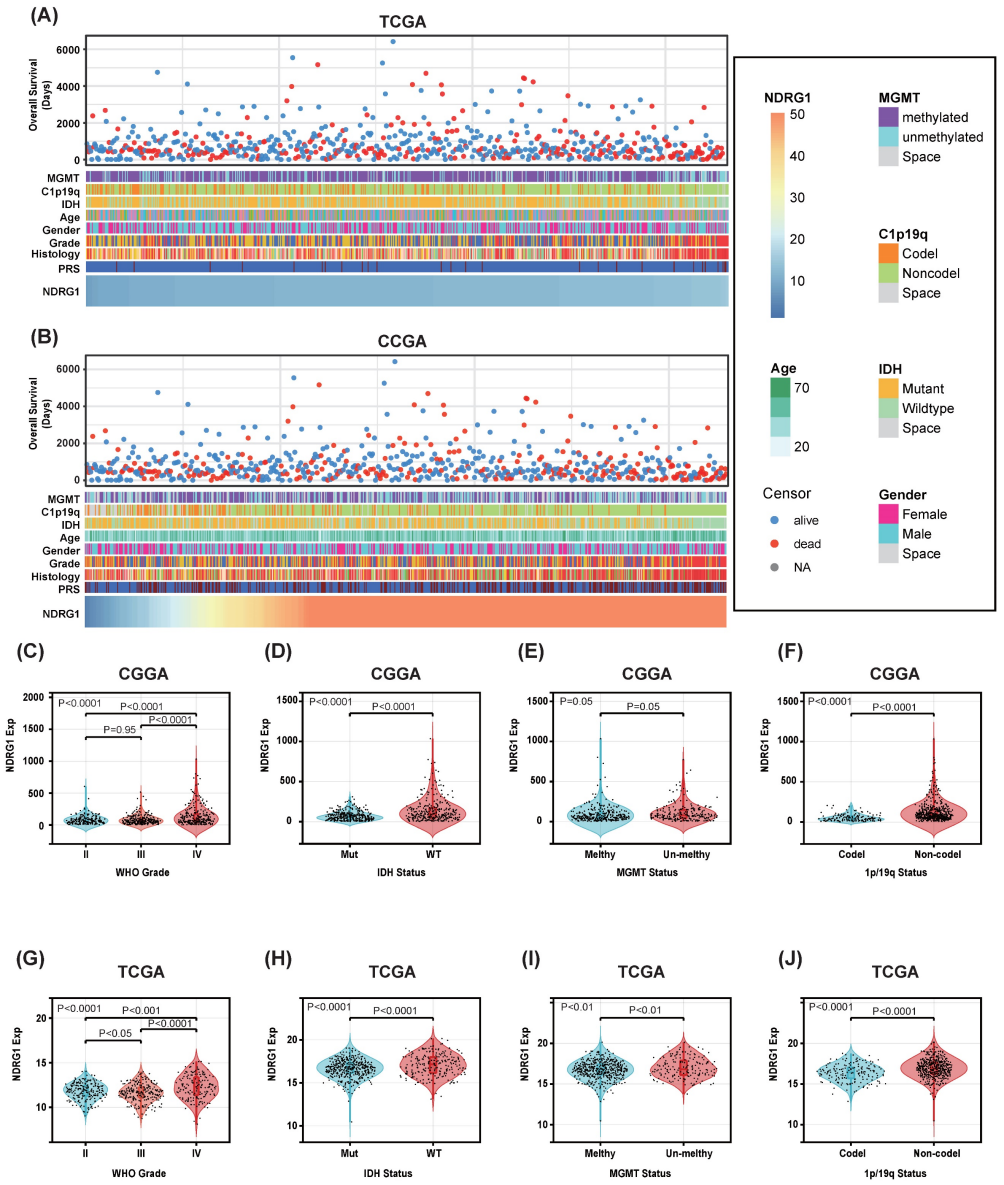
Association between NDRG1 and clinicopathological features of glioma. A Overview of the clinical pathological features of glioma related to NDRG1 in the CGGA database. B Overview of the clinical pathological features of glioma related to NDRG1 in the TCGA database. C and G. Significant increase of NDRG1 in high-grade gliomas in the CGGA and TCGA databases. Significance of the differences tested by Kruskal-Wallis single-factor analysis. D and H Significant increase of NDRG1 in IDH wild-type gliomas in the CGGA and TCGA databases. Significance of the differences tested by unpaired t-test. E and I Significant increase of NDRG1 in neurogliomas with 1p/19q co-deletion. This difference is statistically significant in the TCGA database, while not in the CGGA database. Significance of the differences tested by unpaired t-test. F and J Increase of NDRG1 in MGMT promoter unmethylated neurogliomas in the CGGA and TCGA databases. Significance of the differences tested by unpaired t-test.

Patients with different levels of NDRG1 expression exhibit distinct clinical and pathological features. Increasing levels of NDRG1 are associated with methylated MGMT promoter status, 1p/19q codeletion status, IDH mutation status, and WHO grading in an asymmetric distribution in the CGGA and TCGA datasets (Figure 2A, B). Comparative analysis of different groups of samples was performed. In the CGGA database, NDRG1 is highly enriched in high-grade gliomas and IDH-wildtype gliomas (Figure 2C, D). Additionally, samples without 1p/19q codeletion show higher expression of NDRG1 (Figure 2F). These results were validated in the TCGA database (Figure 2G-J). In the TCGA database, samples without MGMT promoter methylation showed high expression of NDRG1 (Figure 2I). The expression of NDRG1 in the CGGA database also exhibited a similar trend (Figure 2E). Overall, these results suggest that gliomas with higher malignancy levels are enriched with NDRG1.

### The signaling pathways associated with NDRG1 in gliomas

Functional enrichment analysis of NDRG1 was conducted using GSEA on 692 cases from the CGGA database and 659 cases from the TCGA database. The patient information of 692 glioma cases in the CGGA database is shown in Table 2; the information of 659 glioma cases in the TCGA database is shown in Table 3.

**Figure 3:**
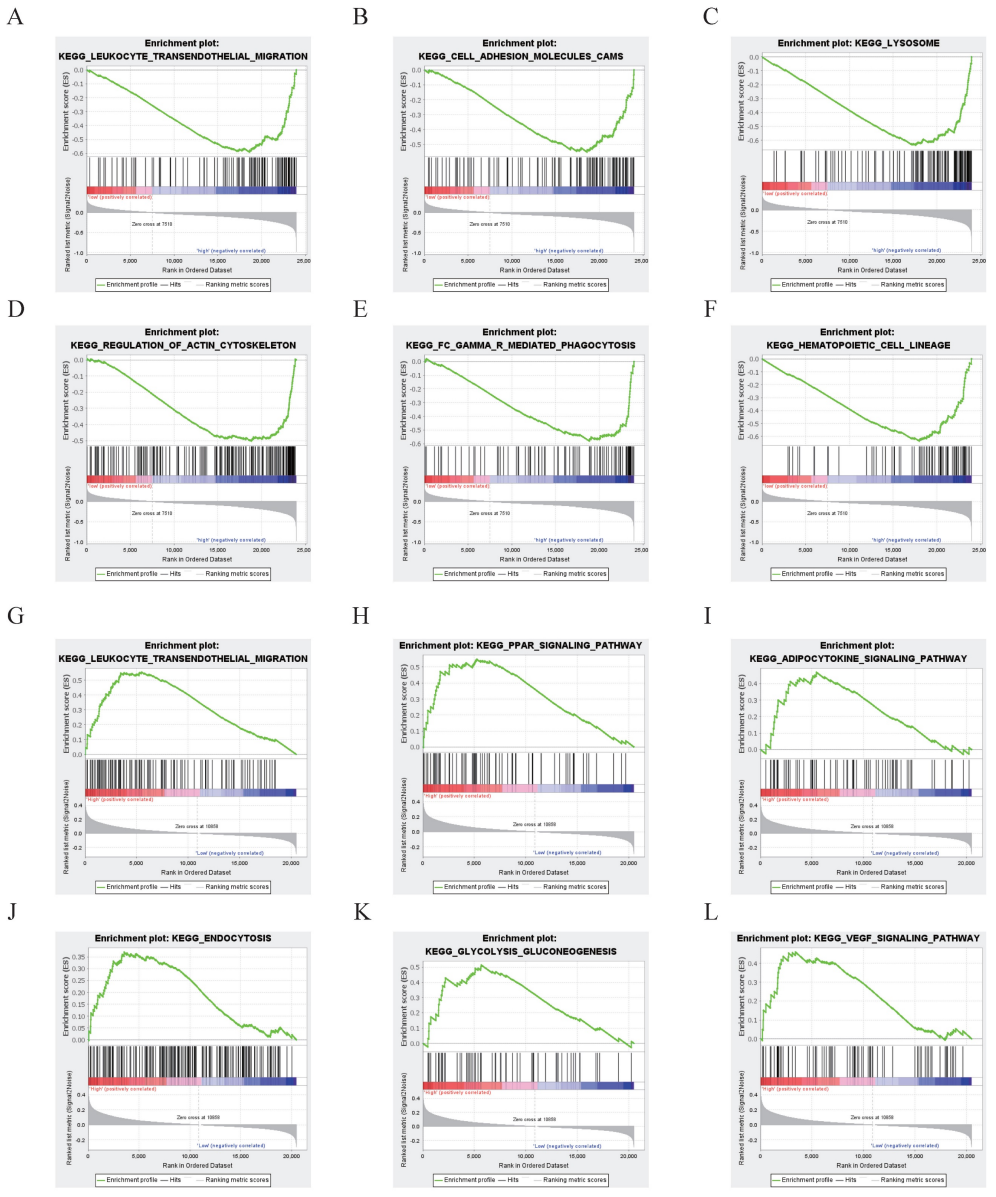
NDRG1 Functional Enrichment Analysis. A-F According to the CGGA database, the gene key set of differential expression with high expression of NDRG1 is mainly focused on signaling pathways such as leukocyte transendothelial migration, cell adhesion molecules, lysosomes, regulation of actin cytoskeleton, γ-mediated phagocytosis, and hematopoietic cell lineage (P<0.05). G-L While in the TCGA database, the key genes with differential expression in the high-expression group of NDRG1 patients are mainly concentrated in signaling pathways such as leukocyte transendothelial migration, PPAR signaling pathway, adipocytokine signaling pathway, endocytosis, glycolysis/gluconeogenesis, and VEGF signaling pathway (P<0.05).

The results showed that the differentially expressed genes in the NDRG1 high-expression group in the CGGA database were enriched in signaling pathways related to leukocyte transendothelial migration, cell adhesion molecules, lysosomes, regulation of the actin cytoskeleton, Fc gamma receptor-mediated phagocytosis, and hematopoietic cell lineage (P<0.05, Figure 3A).

In TCGA glioma patients, the differentially expressed genes in the high NDRG1 expression group are mainly enriched in signaling pathways such as leukocyte transendothelial migration, PPAR signaling pathway, adipocytokine signaling pathway, endocytosis, glycolysis/gluconeogenesis, and VEGF signaling pathway (P<0.05, Figure 3B).

### The expression of NDRG1 is positively correlated with VM and EMT-associated sites

**Figure 4:**
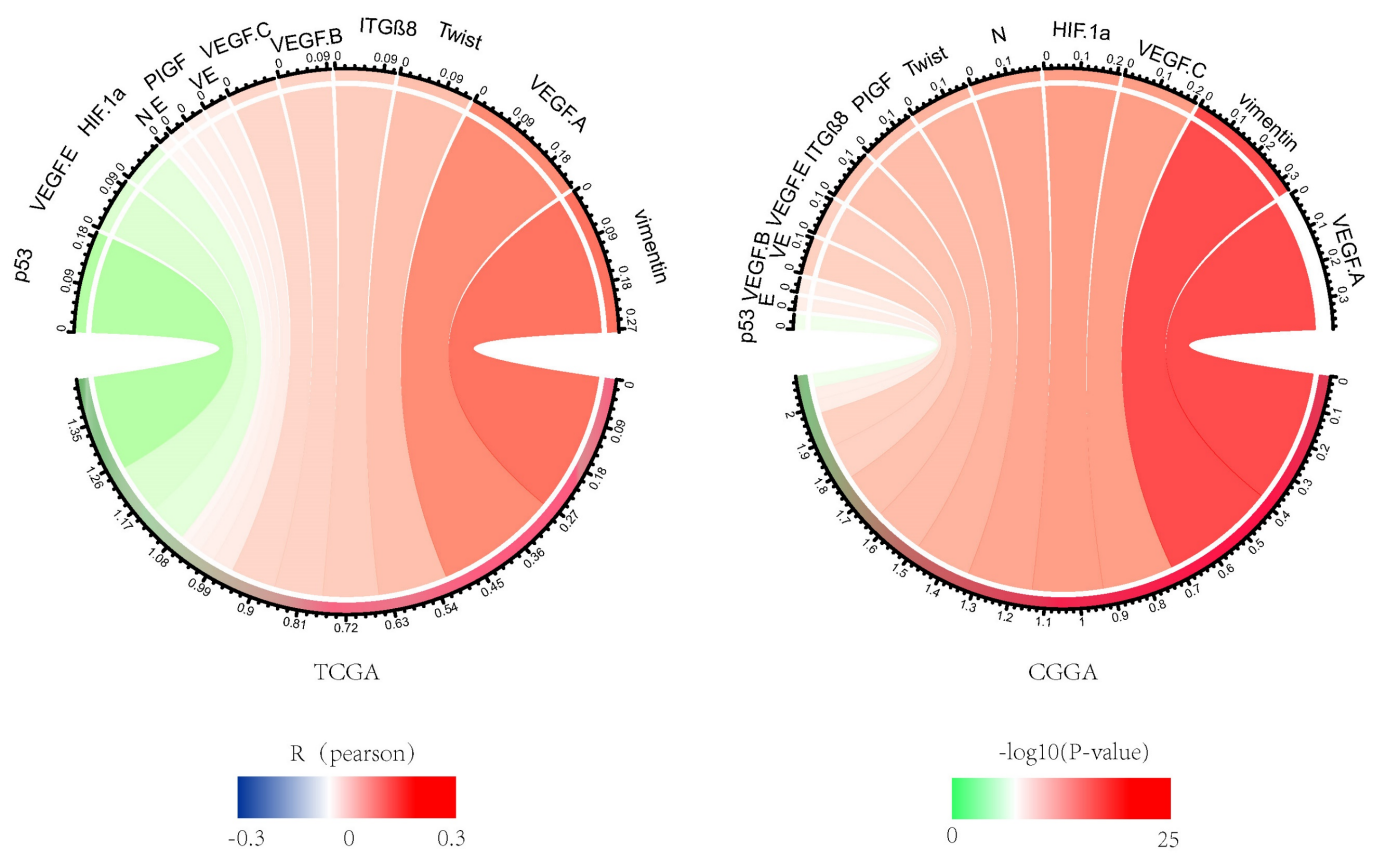
Correlation analysis of NDRG1 with VM and EMT-related sites. The Pearson correlation of NDRG1 with EMT and VM-related sites in CGGA and TCGA databases, where the width represents the R value and the color represents the P value. Green indicates negative correlation, while red indicates positive correlation.

NDRG1 is associated with tumor aggressivity, so we studied VM and EMT related sites, including CDH1, CDH2, VIM, HIF1A, TWIST1, ITGB8, TP53, CDH5, VEGFA, VEGFB, VEGFC, PDGFC, PIGF. NDRG1 showed a strong correlation with EMT and VM in the CGGA and TCGA databases, which lead to glioma growth and invasion (Figure 4).

### NDRG1 is valuable in predicting the prognosis of patients with glioma

**Figure 5:**
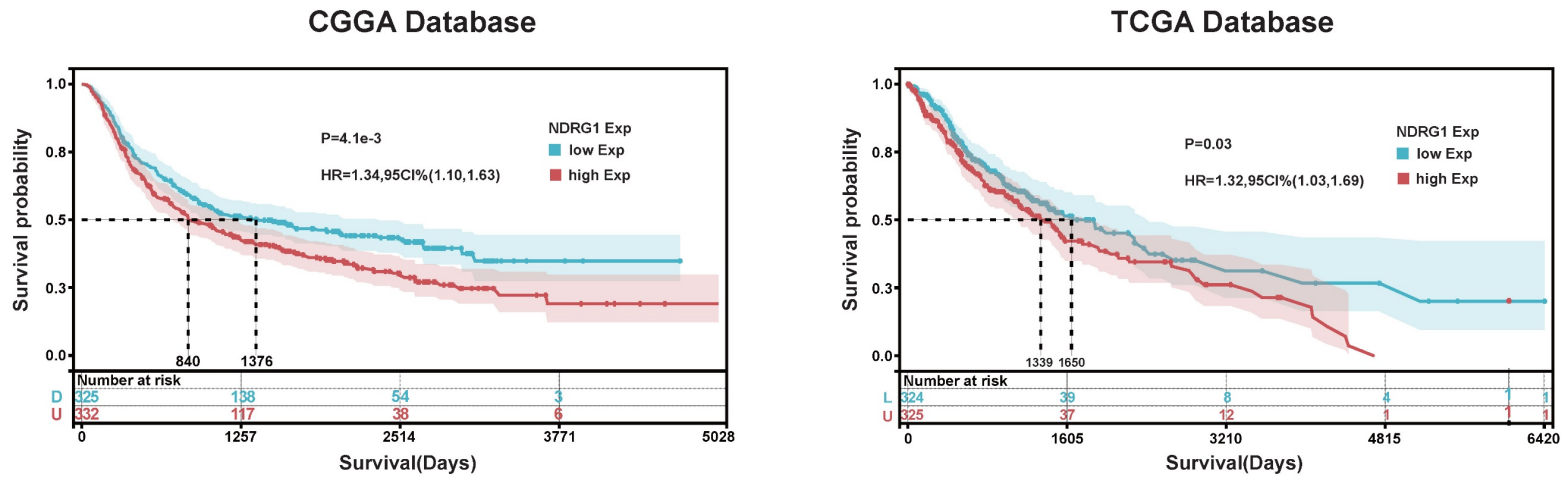
Kaplan- Meier Analysis of NDRG1. Based on Kaplan-Meier analysis of NDRG1 in TCGA and CGGA databases, the cutoff values for Group A and Group B were determined as the median expression of NDRG1. The significance of prognostic value was evaluated using log-rank test.

In order to explore the prognostic value of NDRG1 in glioma patients, we conducted Kaplan-Meier and Cox proportional hazards model analyses based on TCGA and CGGA databases. In the TCGA database, the overall survival of patients with high NDRG1 expression (median survival: 1339 days) was lower than that of patients with low NDRG1 expression (median survival: 1650 days) (Figure 5). Furthermore, the prognostic value of NDRG1 was validated in the CGGA database (Figure 5). In Cox regression analysis, NDRG1 expression was a prognostic factor independent of known prognostic factors, including WHO grade, age at diagnosis, initial recurrence status, and MGMT promoter methylation. These results indicate that NDRG1 is an independent prognostic factor in both TCGA and CGGA databases (Tables 4 and 5).

### Immunohistochemistry

#### The expression of NDRG1 is related to HGG CNS WHO grading

**Figure 6:**
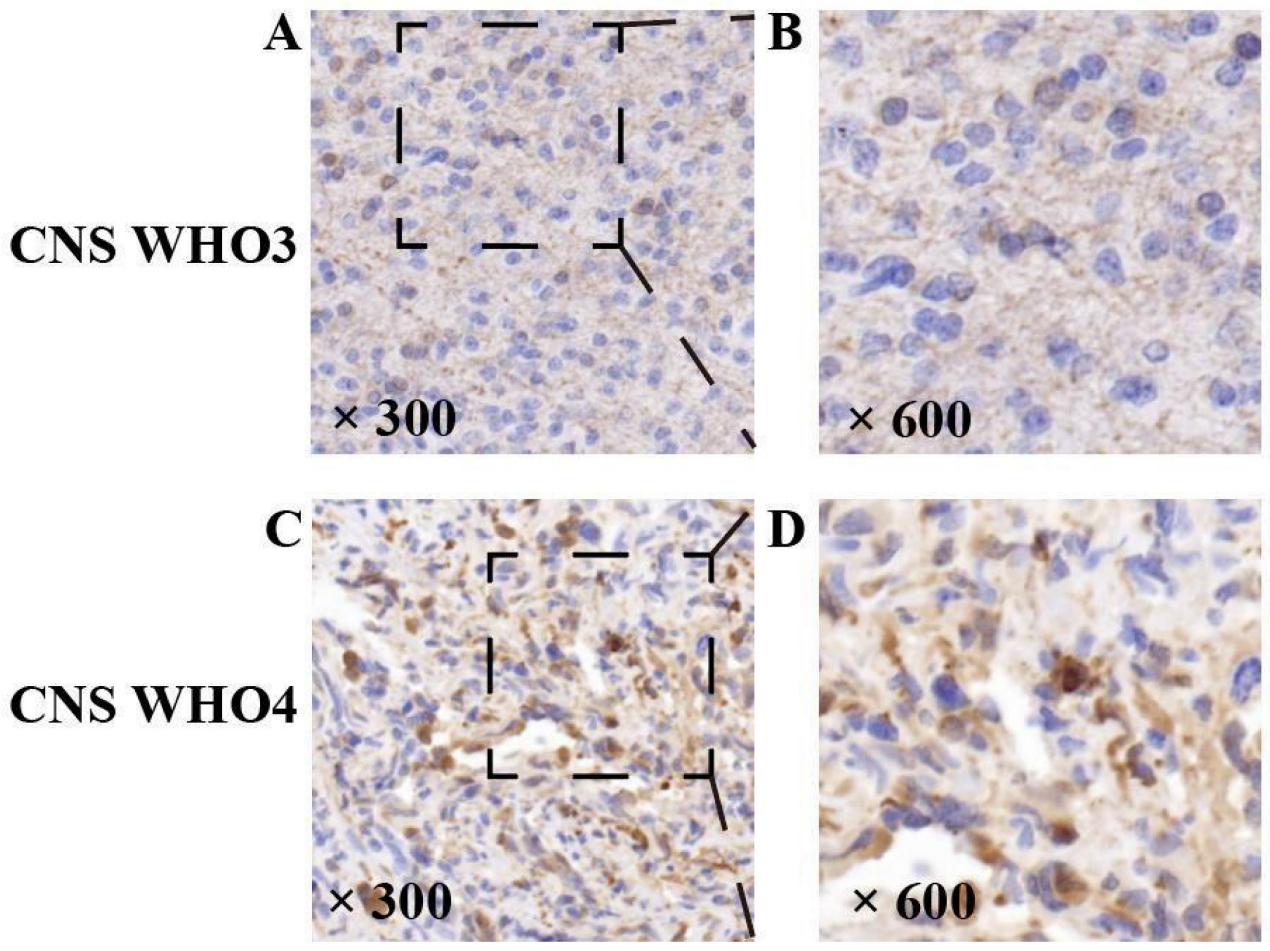
Expression of NDRG1 gene in high-grade gliomas. Representative staining of the NDRG1 gene. The NDRG1 gene expression protein in HGG specimens showed positive immunohistochemical staining, with positive staining visible in the cytoplasm of tumor cells. As the CNSWHO grade increased, the expression of the NDRG1 gene was upregulated (A and B —— CNSWHO grade 3, C and D —— CNSWHO grade 4) (original magnification 300 and 600).

In this experiment, the NDRG1 gene was expressed in all specimens, with its immunoreactivity mainly located in the cytoplasm of tumor cells (Figure 6). To determine the factors influencing HGG CNS WHO grading, we conducted a chi-square test on patient gender, age groups, VM expression, and immunohistochemical scoring levels including NDRG1. The results showed a significant correlation between the immunohistochemical staining score of the NDRG1 gene and HGG CNS WHO grading (P=0.004) (Table 6). Furthermore, to further investigate the relationship between EMT-related proteins including N-cad, E-cad, Vimentin, VEGFa, and HGG malignancy, we conducted a Pearson correlation coefficient analysis. The results indicated that the expression of EMT-related protein Vimentin was positively correlated with the CNS WHO grading of HGG patients (P=0.033) (Table 7).

#### The expression of NDRG1 gene affects tumor VM expression

**Figure 7:**
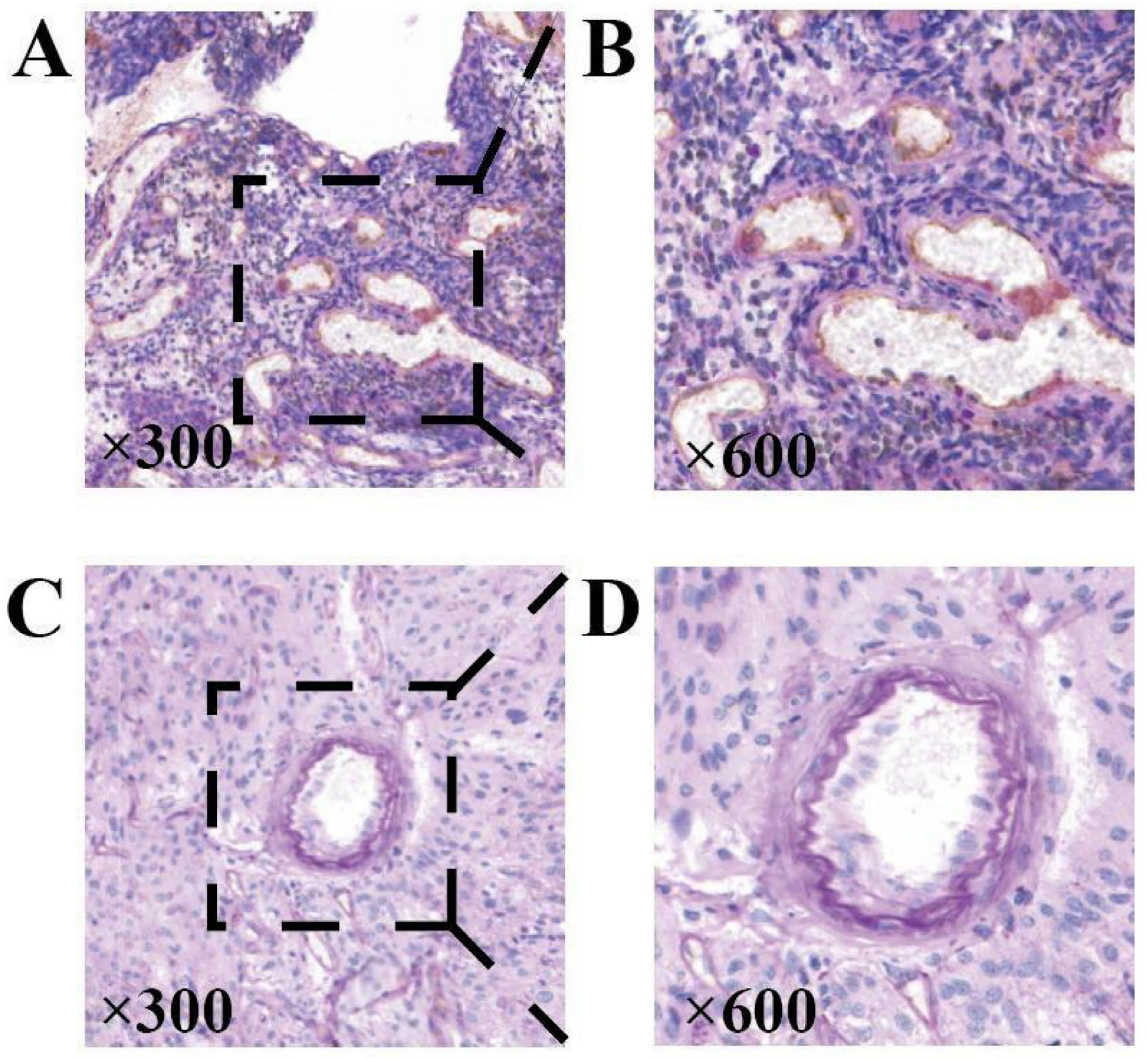
Representative staining of MVD and VM. A and B When measuring the MVD of the sample, classic vascular endothelial immunohistochemical staining can be observed as CD31 positive and PAS staining positive. C and D The typical immunohistochemical characteristics of VM are CD31 negative staining, PAS positive channels, and red blood cells can be found in these channels (original magnification 300x).

**Figure 8:**
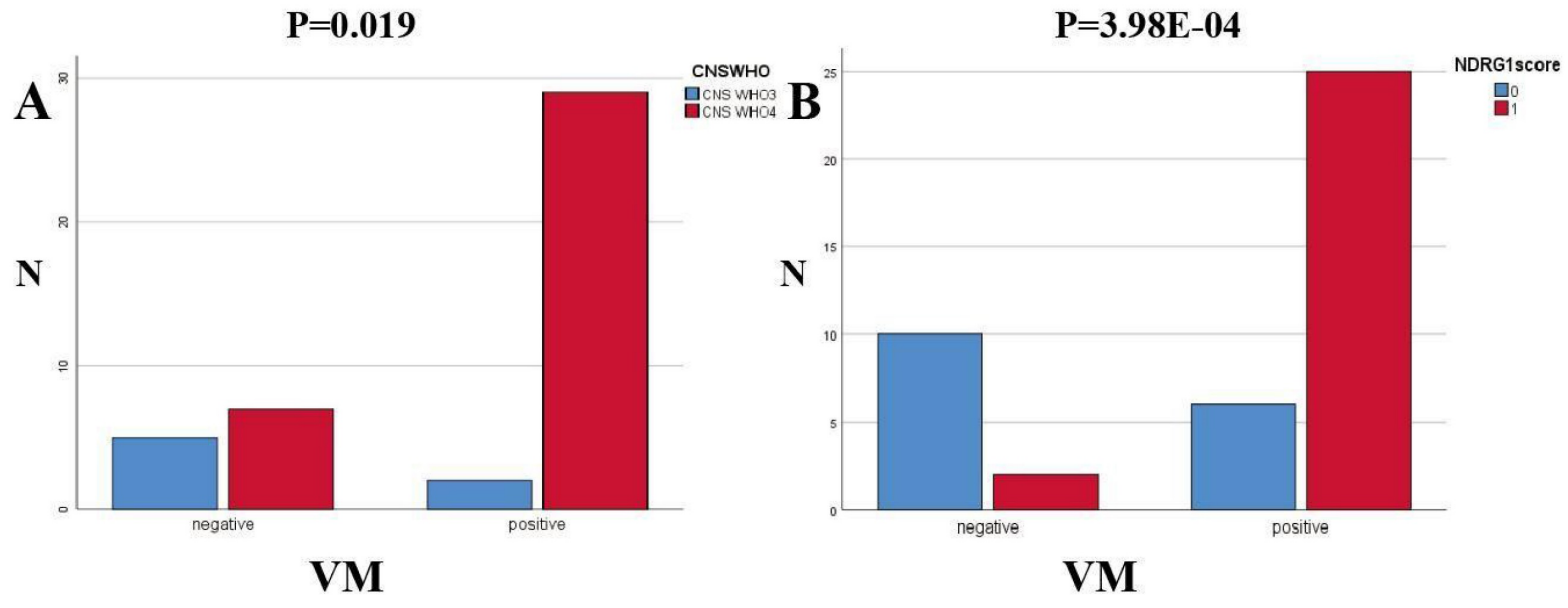
Specimen VM expression. Patients with VM-positive expression and patients with VM-negative expression showed statistically significant differences in NDRG1 gene expression (χ²=9.319, P=0.002) and CNSWHO grading (χ²=4.375, P=0.036), with results showing a positive correlation. Patients with VM-positive expression and patients with VM-negative expression showed statistically significant differences in E-cad expression (χ²=4.142, P=0.042), with results showing a negative correlation.

Key points of judgment under the mirror of VM include: 1. The lumen is surrounded by tumor cells rather than endothelial cells (CD31 negative); 2. Extracellular matrix PAS staining positive; 3. Red blood cells are observed in the lumen (Figure 7). In this study, 31 out of 43 HGG samples (72.09%) were detected with positive expression of VM. Among them, there were 2 cases of CNSWHO grade 3 and 29 cases of CNSWHO grade 4. Moreover, 25 out of the 31 HGG patients with positive VM expression (80.65%) had high expression of NDRG1 gene, while only 2 out of 12 patients with negative VM expression (16.67%) had high expression of NDRG1 gene (Table 8). The expression of VM in patients is statistically significant with the malignancy of tumors (Figure 8A, χ²=5.500, P=0.019), and the difference in NDRG1 gene expression between patients with positive VM expression and patients with negative VM expression is also statistically significant (Figure 8B, χ²=12.542, P=0.0004). The remaining analysis results were not statistically significant (P>0.05).

#### Factors affecting tumor MVD

The calculation method of MVD in 43 specimens in this experiment is to select 5 random fields of view, count the number of CD31-positive microvessels in each random field of view, and then take the average to evaluate the average MVD of the specimens. Subsequently, a one-sample T-test was used to detect the MVD expression in this group of patients. The results showed that the average MVD of 43 patients was (64.87±28.16). Independent sample T-test analysis of the relationship between CNS WHO grading and MVD in HGG patients showed that the average MVD levels of patients in CNS WHO grade 3 group and CNS WHO grade 4 group were (65.8±25.53) and (64.69±28.98) respectively (P>0.05). This means that there was no statistically significant difference in the WHO grading of CNS WHO between MVD and HGG patients. To further investigate the factors influencing MVD, we conducted independent sample t-tests to examine the relationships between MVD and the expression of EMT-related proteins, VEGFa, and NDRG1 gene expression (Table 9). The results showed that NDRG1 gene expression was associated with the MVD status of the samples (χ²=4.743, P=0.035), while the other results were not statistically significant. Subsequently, the research team grouped the patients according to the CNS WHO classification criteria and re-conducted independent sample T-tests related to MVD. In this statistical analysis, it was found that in the CNS WHO grade 4 glioma patient group, the expression of Vimentin protein may be associated with the presence of tumor MVD (χ²=3.504, P=0.070) (Table 10). However, it should be noted that although the P value is 0.07, this value is slightly higher than the commonly accepted significance level of 0.05. Considering the relatively small sample size of this study, caution is advised in interpreting this result and further investigation is warranted. To validate this finding, future research could consider expanding the sample size. Based on the above analysis, the experimental team ultimately concluded that the expression of the NDRG1 gene promotes MVD in HGG patients, and the expression of Vimentin protein may be associated with MVD in CNSWHO grade 4 specimens.

## Discussion

Glioblastoma, as a primary tumor in the human central nervous system, is not only extremely common but also considered one of the most malignant. It is a disease deeply hidden in our nervous system, difficult to grasp and highly destructive^14^. According to recent relevant statistics and epidemiological surveys, HGG, especially GBM, despite the continuous advancement in treatment methods and surgical techniques, still has a relatively short average survival period^15^. This study observed the expression of NDRG1 in 43 patients and found that the expression level of the NDRG1 gene is significantly correlated with the CNS WHO grading of tumors (P=0.004) using relevant statistical methods, strongly suggesting a relationship between this gene and the malignancy of HGG. VM is a special tumor blood supply pattern closely related to tumor growth, invasion, and metastasis. In HGG, enhanced expression of VM implies an increase in tumor blood supply, thus affecting the malignancy of the tumor.

This study distinguished the expression of VM and MVD in the HGG specimens through the CD31/PAS double staining scoring. By analyzing the specimen data using relevant statistical methods, we found a significant correlation between the expression of VM and the expression level of the NDRG1 gene (P=3.98E-04), as well as the tumor CNS WHO grade (P=0.019). Finally, our research team utilized statistical methods such as Pearson correlation analysis and independent sample T-test to study the correlation between MVD in specimens and the expression of related proteins by immunohistochemistry. After statistical analysis, we found that the expression level of the NDRG1 gene was correlated with the MVD values in the specimens (P=0.035).It is worth noting that, after grouping the specimens according to the CNS WHO grading criteria and re-analyzing the data, we found that the independent sample T-test between the MVD values of CNS WHO grade 4 specimens and the expression of Vimentin protein, although not statistically significant (P=0.07), suggests a trend of Vimentin protein expression affecting the MVD values of the specimens. Subsequent studies can further validate this finding by increasing the sample size. MVD is a key indicator for assessing the degree of tumor angiogenesis, and it is closely associated with tumor growth, invasion, and prognosis. In patients with high-grade gliomas (HGG), a high MVD value indicates that the tumor has a stronger angiogenesis capability, thereby increasing the risk of invasion and metastasis. This study found that the NDRG1 gene can influence the expression of VM in specimens, further affecting the CNS WHO grading of HGG patients. Additionally, the NDRG1 gene can regulate tumor MVD, leading to a poor prognosis in HGG patients. In other words, the expression of NDRG1 may be associated with a poorer prognosis in this group of HGG patients. Furthermore, we observed a certain trend in the expression of Vimentin protein and MVD values in CNS WHO grade 4 patients.

After reviewing a large number of literature, we found that the NDRG1 gene is abnormally expressed in various tumors, and related studies have shown that the NDRG1 gene is associated with multiple signaling pathways, including the NF-κB signaling pathway^16^-^18^. NF-κB is a key nuclear transcription factor that typically exists in the cytoplasm of almost all cell types in an inactive form as homodimers or heterodimers^19^. It plays a very important role and is involved in various cellular activities such as activation of immune cells, development of T and B lymphocytes, stress response, and cell apoptosis^20^. Activation of NF-κB is usually triggered by factors such as cellular stress, inflammatory cytokines, growth factors, and ultraviolet radiation. When NF-κB is activated, it translocates from the cytoplasm to the nucleus and binds to specific DNA sequences (κB sites) to form a DNA/NF-κB complex. This complex initiates the transcription of downstream DNA sequences and the synthesis of corresponding proteins, thereby exerting various biological functions. In the NF-κB signaling pathway, NF-κB typically forms a complex with its inhibitory protein IκBs, existing in an inactive form in the cytoplasm. When cells are exposed to various activating factors, IκBs are phosphorylated and degraded, leading to the release and activation of NF-κB. Studies have found that NDRG1 can inhibit the nuclear translocation and transcriptional activity of NF-κB by inhibiting the activity of IKKβ (a key kinase in the NF-κB signaling pathway). This inhibitory effect has been validated in various tumor cells, indicating that NDRG1 may participate in tumor immune evasion and inflammation by regulating the NF-κB pathway^21^-^23^. Literature indicates that NF-κB is often in an activated state in gliomas, and this activation is closely associated with the malignancy, invasiveness, and poor prognosis of gliomas. NF-κB promotes the epithelial-mesenchymal transition (EMT) process of glioma cells by regulating the expression of EMT-related proteins such as Snail, Slug, and Twist. These transcription factors can enhance the expression of Vimentin, thereby promoting the migration and invasion of glioma cells^24^. Meanwhile, the NF-κB pathway is closely related to angiogenesis and tumor progression. NF-κB can regulate the expression of various genes and proteins related to angiogenesis, thus affecting the formation and density of microvessels^25^, ^26^. In addition, the activation of NF-κB can promote the proliferation, migration, and invasion of tumor cells, processes that are closely associated with tumor angiogenesis and increased MVD^27^-^29^. These research findings well explain why there is a trend of association between the expression of Vimentin protein and MVD in CNS WHO4 grade patients.

In conclusion, our data show that the expression level of the NDRG1 gene is associated with the CNS WHO grade of HGG patients. High expression levels of the NDRG1 gene can identify HGG patients with poor prognosis, specifically CNS WHO grade 4 HGG patients. Therefore, the NDRG1 gene can be defined as a potential predictive factor for poor prognosis in HGG patients. The expression of the NDRG1 gene in HGG is related to MVD and VM, suggesting that the NDRG1 gene may be an important target for HGG treatment.

## Figures and Tables

**Table 1:** General clinical data of patients

		CNSWHO		Total
		CNS WHO3	CNS WHO4	(n=43) (%)
Age, years	<60	5	16	21 (48.8)
	>60	2	20	22 (51.2)
Sex	Female	1	11	12 (27.9)
	Male	6	25	31 (72.1)

**Table 2:** Clinical information of 692 patients with glioma in CGGA database

		Alive (n=266)	Dead (n=397)	Asymptomatic survival (n=29)	Total (n=692)
Gender, n (%)	Female	121 (17.5)	163 (23.6)	10 (1.4)	294 (42.5)
	Male	145 (21)	234 (33.8)	19 (2.7)	398 (57.5)
Age	Mean	40.3	45.5	41.1	43.3
	Median	40	46	39	43
WHO, n (%)	II	122 (17.6)	54 (7.8)	12 (1.7)	188 (27.2)
	III	104 (15)	145 (21)	6 (0.9)	255 (36.8)
	IV	40 (5.8)	198 (28.6)	11 (1.6)	249 (36)
Primary/Recurrent Status, n (%)	Primary	196 (28.3)	210 (30.3)	16 (2.3)	422 (61)
	Recurrent	70 (10.1)	187 (27)	13 (1.9)	270 (39)

**Table 3:** Clinical information of 659 patients with glioma in TCGA database

		Alive (n=401)	Dead (n=252)	Asymptomatic survival (n=6)	Total (n=659)
Gender, n (%)	Female	178 (27)	99 (15)	3 (0.4)	277 (42)
	Male	223 (33.9)	153 (23.2)	3 (0.4)	379 (57.5)
Age	Mean	42.3	54.4		46.9
	Median	40	57		39
WHO, n (%)	II	201 (30.5)	37 (5.6)		238 (36.1)
	III	171 (26)	87 (13.2)	3 (0.4)	261 (39.6)
	IV	29 (4.4)	128 (19.4)	3 (0.4)	160 (24.2)
Primary/Recurrent Status, n (%)	Primary	394 (59.8)	232 (35.2)	6 (0.9)	632 (95.9)
	Recurrent	7 (1.1)	20 (3.0)	0	27 (4.1)

**Table 4:** Univariate and multivariate analysis of prognostic parameters of total OS in TCGA database

	Single Factor Analysis		Multi-Factor Analysis	
	95% CI	P	95% CI	P
NDRG1	1.306 (1.150-1.483)	3.78E-05	1.140 (1.011-1.286)	0.0328
WHO grading system	5.887 (4.106-8.440)	5.22E-22	4.084 (2.802-5.951)	2.41E-13
Age	4.307 (3.120-5.946)	6.98E-19	3.901 (2.713-5.608)	2.01E-13
Primary/Recurrent Status	1.469 (0.929-2.324)	0.1		
MGMT	0.293 (0.222-0.385)	1.4E-18	0.350 (0.263-0.467)	7.46E-13

**Table 5:** Univariate and multivariate analysis of prognostic parameters of total survival time (OS) in CGGA

	Single Factor Analysis		Multi-Factor Analysis	
	95% CI	P	95% CI	P
NDRG1	1.002 (1.001-1.002)	9.924E-07	1.001 (1.000-1.002)	0.03
WHO grading system	3.896 (2.894-5.246)	3.094E-19	3.216 (2.297-4.503)	1.02E-11
Age	1.636 (1.321-2.027)	6.55E-06	1.600 (1.261-2.031)	1.11E-04
Primary/Recurrent Status	2.182 (1.785-2.667)	2.597E-14	2.085 (1.666-2.610)	1.35E-10
MGMT	0.795 (0.639-0.990)	0.041	0.760 (0.610-0.947)	0.015

**Table 6:** Factors influencing the CNS WHO grading of HGG patients.

		CNSWHO		χ²	P
		CNS WHO3	CNS WHO4		
Sex	Male	6	25	0.771	0.380
	Female	1	11		
Age,years	<60	5	16	1.708	0.191
	>60	2	20		
NDRG1 score	Low	6	10	8.420	0.004
	High	1	26		

**Table 7:** EMT-related protein immunohistochemical scoring in CNS tumors related analysis of WHO grading.

		MVD	Ncad	Ecad	VEGFa	Vimentin	CNS WHO
MVD	r	1	.002	-.188	-.116	.024	-.015
	P		.990	.227	.460	.878	.925
Ncad	r	-0.02	1	.184	.096	-.088	.067
	P	.990		.239	.540	.575	.669
Ecad	r	-.188	.184	1	.257	.081	.132
	P	.227	.239		.097	.604	.399
VEGFa	r	-.116	.096	.257	1	.036	.008
	P	.460	.540	.097		.819	.959
Vimentin	r	.024	-.088	.081	.036	1	.326
	P	.878	.575	.604	.819		.033

**Table 8:** Chi-square test for VM expression in cancer patients

		VM		Total	χ²	P
		negative	positive			
CNS WHO	CNS WHO3	5	2	7	5.500	0.019
	CNS WHO4	7	29	36		
NDRG1 score	Low	10	6	16	12.542	3.98E-04
	High	2	25	27		
N-cad score	Low	10	28	38	0.012	0.912
	High	2	3	5		
E-cad score	Low	6	23	29	1.336	0.248
	High	6	8	14		
VEGFA score	Low	9	19	28	0.240	0.625
	High	3	12	15		
Vimentin score	Low	10	18	28	1.447	0.229
	High	2	13	15		
n=43		n=12	n=31			

**Table 9:** Independent sample T-test related to specimen MVD

		MVD				
		n	Mean	SD	χ²	P
CNS WHO	3	7	65.80	25.53	0.889	0.351
	4	36	64.69	28.98		
NDRG1 score	Low	16	61.51	21.12	4.743	0.035
	High	27	66.86	31.82		
N-cad score	Low	38	64.49	29.45	1.356	0.251
	High	5	67.72	17.17		
E-cad score	Low	29	68.08	29.78	0.997	0.324
	High	14	58.23	24.12		
Vimentin score	Low	28	67.76	31.24	2.945	0.094
	High	15	59.48	21.19		
VEGFA score	Low	28	66.26	27.59	0.960	0.333
	High	15	62.27	30.01		

**Table 10:** Independent sample T-test related to specimen MVD.

CNS WHO	Vimentin score	MVD				
		n	Mean	SD	χ²	P
CNS WHO3	Low	6	66.0333	27.96352		
	High	1	64.4000	/		
CNS WHO4	Low	22	68.2273	32.6780	3.504	0.070
	High	14	59.1286	21.9404		
